# Effects of exercise training on circulating levels of Dickkpof-1 and secreted frizzled-related protein-1 in breast cancer survivors: A pilot single-blind randomized controlled trial

**DOI:** 10.1371/journal.pone.0171771

**Published:** 2017-02-08

**Authors:** Tae Ho Kim, Jae Seung Chang, Kyu-Sang Park, Jeeyeon Park, Nahyun Kim, Jong In Lee, In Deok Kong

**Affiliations:** 1 Department of Physiology, Wonju College of Medicine, Yonsei University, Wonju, Republic of Korea; 2 Yonsei Institute of Sports Science & Exercise Medicine, Wonju, Republic of Korea; 3 Department of Nursing Science, Kyungsung University, Busan, Republic of Korea; 4 Department of Basic Nursing Science, Keimyung University College of Nursing, Daegu, Republic of Korea; 5 Department of Hematology-Oncology, Wonju College of Medicine, Yonsei University, Wonju, Republic of Korea; Fondazione IRCCS Istituto Nazionale dei Tumori, ITALY

## Abstract

**Background:**

Wingless and integration site growth factor (Wnt) signaling is a tumorigenesis-related signaling pathway. Dickkpof-1 (DKK1) and secreted frizzled-related protein-1 (SFRP1) are endogenous negative regulators of Wnt/β-catenin signaling. Accumulating evidence indicates that higher serum levels of DKK1 are correlated with poor prognosis of various types of cancer. Here, we investigated whether exercise training causes changes in the serum levels of DKK1 and SFRP1 in patients with breast cancer.

**Methods:**

Twenty-four breast cancer survivors, after chemo- or radiotherapy, participated in this single-blind randomized, controlled pilot study. Subjects were randomized to either an exercise program or a control group for 12 weeks and completed pre- and post-training tests for health-related fitness and body composition as well as blood biomarkers. The serum levels of DKK1 and SFRP1 were measured using enzyme-linked immunosorbent assay as the primary outcome.

**Results:**

Exercise training for 12 weeks remarkably increased muscle strength, endurance, and flexibility and decreased body fat percentage, waist circumference, and visceral fat area (all *p* < 0.05). Exercise training lowered serum insulin levels and leptin/adiponectin ratios (all *p* < 0.05). The levels of DKK1 and SFRP1 were also significantly decreased by exercise training in breast cancer survivors (all *p* < 0.01).

**Conclusions:**

Our results indicate that DKK1 and SFRP1 may be potentially useful biomarkers for evaluating the beneficial effects of long-term exercise on physical fitness and metabolism as well as the prognosis of patients with cancer.

**Trial registration:**

ClinicalTrials.gov NCT02895178

## Introduction

Patients and survivors of breast cancer present impaired physical fitness and various complications including acute and chronic pain, severe fatigue, limited range of motion, and bone loss attributable to anti-cancer treatments [1,2]. Therefore, regular exercise during and following cancer treatments has been recommended to enhance physical capabilities and reduce the severity of side-effects of treatments, leading to an improved quality of life [2–4]. Despite the known general benefits to cancer patients, the effects of exercise on the initiation and progression of the tumor itself remain unclear.

Wingless and integration site growth factor (Wnt) signaling is one of the major tumorigenesis-related signaling pathways [5]. Secreted Wnt and its downstream effectors regulate critical processes during tumor growth and metastasis [6]. Additionally, Wnt mutations are associated with breast cancer and other common types of cancer [5,6]. Dysregulated Wnt signaling has been detected in carcinogenesis of the mammary gland [7–9]. The molecular mechanisms of Wnt signaling are categorized into β-catenin-dependent (canonical) and -independent (non-canonical) pathways [10,11]. The canonical pathway is activated by the binding of Wnt ligands to Frizzled receptors and low-density lipoprotein receptor-related protein 5 and 6 (LRP5/6) on the cell membrane, promoting the stabilization and transcriptional activity of β-catenin [9,12]. Non-canonical Wnt signaling is composed of two well-characterized pathways: the planar cell polarity pathway and the Wnt/Ca^2+^ pathway [13–15].

For the past two decades, a number of endogenous modulators of Wnt signaling have been identified. Among them, Dickkpof-1 (DKK1) is a soluble inhibitor of the Wnt signaling pathway, which acts by binding to LRP5/6 and Kremen protein to induce endocytosis, causing proteosomal degradation of β-catenin [9,16,17]. Secreted frizzled-related protein-1 (SFRP1) blocks Wnt signaling by binding to Wnt ligands or Frizzled receptors [9]. DKK1 can inhibit only the canonical pathway, whereas SFRP1 can antagonize both canonical and non-canonical Wnt signaling [18–20]. Paradoxically, DKK1 levels were found to be markedly increased in patients with breast cancer compared with both, women in complete remission and healthy controls [21,22]. Higher serum levels of DKK1 were correlated with bone metastasis of breast cancer and its mortality [16,17]. A correlation between high DKK1 and poor prognosis was also observed in serological samples from patients with pancreatic, prostate, stomach, liver, and lung cancers regardless of the presence of metastatic dissemination to the bone [23–25]. Furthermore, inhibition of DKK1 by neutralization decreased tumor growth [25]. Therefore, the regulation of DKK1 may be a therapeutic target in the development of anti-cancer therapy.

A recent study reported that serum DKK1 levels were significantly decreased after participation in an ultradistance marathon [26]. An animal study also showed that sedentary conditions elevated DKK1 expression in brain tissue, indirectly showing that physical activity downregulates DKK1 [27]. In this study, we detected reduced serum levels of DKK1 and SFRP1 in breast cancer survivors following a long-term (12-week) exercise program. These changes were accompanied by improved physical fitness and biomarker levels related to metabolic conditions, indicating the possible involvement of Wnt antagonists in the beneficial effects of exercise.

## Methods

The supporting study protocol and CONSORT checklist are available as supporting information; see S1 and S2 Texts (original language and English versions) and S1 Table.

### Study participants

Survivors of breast cancer who visited the hemato-oncology center of Wonju Severance Christian Hospital were recruited between June 1 and December 31, 2014. They were eligible to participate in this study if they met the following criteria, with medical clearance from their oncologist: (1) diagnosed with stage I–III breast cancer, (2) ≥ 6 months after treatments with radio- and/or chemotherapy subsequent to surgery, (3) absence of metastatic diseases and other cancers, (4) < 60 min per week of physical activity including resistance exercise in the past 6 months, (5) absence of cardiovascular and respiratory diseases, and (6) no contraindicated medications and comorbidities that prohibit participation in a moderate exercise program. After the intervention period of 12 weeks, the study participants were followed-up between September 16, 2014 and April 30, 2015. A total of 45 subjects were included in the final analysis (exercise group, n = 11; non-exercise control group, n = 13; age-matched healthy women, n = 21). All procedures and informed consent document were reviewed and approved by the Medical Ethics Committee of Yonsei University Wonju College of Medicine, Korea (YWMR-14-0-042; approval date: May 23, 2014). Written informed consent was obtained from all participants included in the study. The study was conducted in accordance with the Declaration of Helsinki principles.

### Design and procedures

Treatment history and menstrual status were obtained from the clinical reports of the hemato-oncology center with the consent of participants. Demographic characteristics were collected through self-reports (Table 1). Measurements were conducted at baseline and after 12 weeks exercise training and included anthropometry, body composition, health–related fitness levels, and a blood sample. As this was a randomized pilot study, a sample size calculation was not performed. Participant recruitment and enrollment began after ethical approval, but the protocol of this pilot trial was retrospectively registered on ClinicalTrials. gov (identification number: NCT02895178), as the authors were unaware of the journal requirements. The authors confirm that all ongoing and related trials for this exercise intervention are registered.

**Table 1:** Baseline demographic characteristics.

Variable	Control group	Exercise group	Total participants
Age (years)*	49.3 (4.8)	56.0 (6.5)	52.4 (6.5)
Height (cm)*	156.1 (3.7)	157.0 (5.0)	156.5 (4.3)
Weight (kg)*	61.0 (11.9)	59.1 (7.1)	60.2 (9.8)
BMI (kg/m^2^)*	25.0 (4.7)	23.9 (2.7)	24.5 (3.9)
Body fat (%)*	34.6 (7.4)	36.1 (4.6)	35.3 (6.2)
Married^†^	13 (100)	11 (100)	24 (100)
Graduate university^†^	5 (38.4)	4 (36.3)	9 (37.5)
Current smoker^†^	0 (0)	0 (0)	0 (0)
Postmenopausal^†^	13 (100)	10 (91)	23 (95.8)
Mastectomy^†^	2 (15.4)	2 (18.2)	4 (16.7)
Lumpectomy^†^	11 (84.6)	9 (81.8)	20 (83.3)
Radiotherapy^†^	12 (92.3)	10 (90.9)	22 (91.6)
Chemotherapy^†^	12 (92.3)	9 (81.8)	21 (87.5)

Data are presented as *mean (standard deviation) or ^†^n (%).

### Randomization and blinding

Following baseline assessments, thirty participants were randomly assigned to either an exercise intervention group or a control group using a sealed, computer random number generator with an allocation ratio of 1 to 1. Four research staff members who were unaware of group assignment performed all outcome assessments. The statistician was unaware of treatment allocation until completion of the statistical analyses. Participants were not blinded to their assignment but were unaware of main outcome measures and were instructed to avoid mentioning anything regarding their study experience to the assessors.

### Outcome measures

The primary outcome measures were serum levels of DKK1 and SFRP1. The secondary outcome measures included anthropometric and body composition indices, health-related fitness parameters, and serological biomarkers.

#### Biomarker measurements

Blood samples were drawn from the antecubital vein and collected in serum separation tubes and the tubes were centrifuged at 3000 rpm (1,000 × *g*) for 10 min. Serum samples were collected and immediately stored at -80°C until analysis. Commercially available enzyme linked immune sorbent assay kits were used to measure the serum levels of leptin (DLP00, R&D Systems, Minneapolis, MN, USA), adiponectin (DRP300), DKK1 (DKK100), and SFRP1 (SEF880Hu, USCN Life Science, Wuhan, China). Serum insulin and high-sensitivity C-reactive protein were measured by electrochemiluminescence immunoassay (Roche cobas 8000-e602 module, Roche Diagnostics, Basel, Switzerland) and latex-enhanced immunoturbidimetric assay (Roche-Hitachi cobas c system, Roche Diagnostics), respectively.

#### Anthropometry and body composition measurements

\Body weight and height were measured to the nearest 0.1 kg and 0.1 cm, respectively, and then body mass index was calculated as body weight divided by height in meters squared (kg/m^2^). Waist circumference was measured at the midpoint between the lower rib margin and iliac crest and was expressed in centimeters. Body fat percentage, visceral fat area, and arm circumferences were measured by multifrequency bioelectrical impedance analysis (Inbody 720, Biospace, Centennial, CO, USA).

#### Health-related physical fitness measurements

Subjects were evaluated for health-related components of fitness such as standing long jump, handgrip strength, sit-up, 10-m shuttle run, 20-m pacer, and sit and reach. The test parameters indicate individual abilities of physical fitness regarding muscular power, strength and endurance, agility, aerobic capacity, and flexibility.

### Exercise intervention group

The exercise training program was designed following the American College of Sports Medicine’s guide to exercise and cancer survivorship (exercise intensity, frequency, time, and type), and was performed at least thrice weekly for 12 weeks, under the direct supervision of exercise physiologists [28]. Exercise training was conducted with the Borg’s ratings of perceived exertion (RPE) scale within the range of RPE 11–13 that was gradually and moderately increased at 4-week intervals until reaching a rating of 13–15 [29]. Each session began with a warm-up consisting whole body stretching and flexibility exercises for shoulder muscle stiffness such as finger climbing, shoulder glides (inferior, anterior, and posterior), and pendulum exercises for 10 min. The exercise program incorporated step aerobics on 17-cm (6.7-inch) platforms for 20 min followed by the strength training using body weight and elastic bands consisting of shoulder press, black burn exercise, wall push-up, biceps curl-up, plank exercise, leg bridge, squat, and calf raise for 20 min. The strength training was designed to begin with one set for the first two weeks, and the set number was increased every two weeks to finally achieve three sets of each exercise performing 12‒16 repetitions to volitional fatigue per set. The exercise intensity and the resistance of elastic band were progressively increased to maintain this range of repetition. At the end of the session, subjects performed cool-down involving easy walking and stretching exercises for 10 min.

### Control group

Subjects in the control group were instructed to maintain their routine physical activities and not to participate any new exercise programs during the 12-weeks study period. Afterward, the subjects who completed both pre- and post-test received an opportunity to participate in the same exercise program that intervention group had performed.

### Age-matched healthy women

Twenty-one age-matched healthy women (mean ± SD, 51.7 ± 5.9 years) were included in this study, in order to compare the basal serum levels of DKK1 between breast cancer survivors and healthy women without a history of breast cancer. The average BMI and body fat percentage of the healthy volunteers were 23.8 ± 2.5 kg/m^2^ and 33.6 ± 6.7%, respectively. All healthy volunteers were married, and non-smokers. Most of the demographic characteristics of the subject corresponded closely to those of breast cancer survivors.

### Data analysis

All data were analyzed using SPSS 22.0 software (SPSS, Inc., Chicago, IL, USA). Descriptive statistics were calculated to identify the means and standard deviations or standard errors of mean (SEM). A Fisher’s exact test was used to assess the homogeneity of age distribution between the two groups. Paired *t*-test or Wilcoxon signed-rank test were used to compare the levels of health-related fitness, body composition, and serum biomarkers between before and after training. Statistical significance was set at *p* < 0.05.

## Results

**Fig 1:**
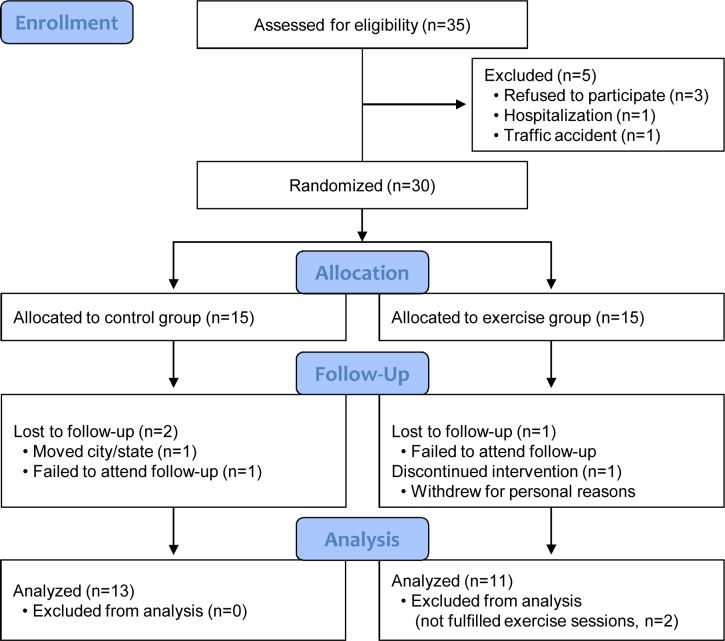
Study flow diagram. This flow diagram shows the study protocol and sequence of events in this study.

The study flow diagram for the recruitment, participation, and dropout of breast cancer survivors is presented in Fig 1. The final dropout rate after 12 weeks in the exercise group was 26% and that in the control group was 13%. Of the 30 women who were eligible for inclusion in this study, six were excluded from final analysis. The reasons for non-inclusion in the analysis were as follows: three women were absent at post-test, two did not fulfill the required exercise sessions of at least three times per week, and one withdrew from the exercise program for personal reasons.

The Fisher’s exact test indicated no significant difference in age distribution between exercise group and control group (*p* = 0.150). Exercise training for 12 weeks resulted in noticeable changes in anthropometry and body composition. Waist circumference was decreased by 2.8% (*p* = 0.041) and total body fat and visceral fat area were reduced by 5.5% (*p* = 0.005) and 5% (*p* = 0.010), respectively, through exercise training (Table 2). Exercise training decreased right (*p* = 0.027) and left (*p* = 0.056) arm circumferences, reflecting a lymphedema status (Table 2). Similarly, overall health-related physical fitness was also improved in the exercise group, but no changes occurred in the non-exercise control group. Muscular strength measured by a handheld dynamometer and its relative value (handgrip strength to weight ratio) were significantly increased by 14% (*p* = 0.043) and 16% (*p* = 0.039), respectively, through the exercise training program (Table 2). In addition, muscular endurance measured in the sit-up test (*p* = 0.025) and flexibility assessed in the sit and reach (*p* = 0.010) tests were enhanced through exercise training (Table 2). Muscular power, agility, and aerobic capacity remained unchanged in both groups after 12 weeks (Table 2).

**Table 2:** Changes in anthropometrics, body composition, health-related fitness, and blood variables between pre- and post-exercise training.

	Control group			Exercise group		
Variables	Pre	Post	*p*-value	Pre	Post	*p*-value
Anthropometry						
Weight (kg)	61.0 (11.9)	61.6 (12.1)	0.490	59.2 (7.1)	58.4 (8.0)	0.150
Waist circumference (cm)	82.0 (13.7)	81.4 (15.5)	0.225	81.6 (7.1)	79.4 (6.7)	0.041
Right-arm circumference (cm)	30.4 (4.1)	30.6 (4.0)	0.205	29.2 (2.0)	28.7 (1.7)	0.027
Left-arm circumference(cm)	30.2 (3.9)	30.4 (4.0)	0.073	29.1 (2.0)	28.7 (1.7)	0.056
Body composition						
Body fat (%)	34.6 (7.4)	36.2 (6.8)	0.074	36.1 (4.6)	34.1 (5.5)	0.005
Visceral fat area (cm^2^)	88.7 (30.4)	90.3 (32.3)	0.453	86.5 (20.4)	82.2 (21.4)	0.010
Health-related fitness						
Handgrip strength (kg)	25.2 (3.3)	24.9 (4.4)	0.701	22.3 (5.1)	25.4 (3.1)	0.043
Relative handgrip strength ratio	42.2 (6.7)	41.7 (8.3)	0.647	37.8 (1.4)	43.8 (8.9)	0.039
Sit-up (n)	13.2 (7.7)	13.6 (8.3)	0.174	15.9 (9.1)	16.5 (11.9)	0.025
Sit and reach (cm)	14.9 (6.2)	13.6 (6.2)	0.304	10.2 (8.4)	12.7 (7.4)	0.010
Standing long jump (cm)	126.5 (21.2)	127.0 (21.7)	0.056	114.5 (20.3)	110.7 (13.3)	0.355
10 meter shuttle run (sec)	16.3 (4.2)	16.8 (2.7)	0.062	15.2 (0.9)	16.4 (4.6)	0.216
20 meter pacer (n)	13.2 (5.8)	10.9 (5.9)	0.843	10.5 (6.3)	12.1 (4.7)	0.108
Blood variables						
Insulin (*u*IU)	10.4 (7.5)	11.2 (9.5)	0.734	11.2 (6.1)	7.6 (3.0)	0.018
hs-CRP (mg/mL)	1.60 (2.89)	1.43 (1.53)	0.431	1.18 (1.37)	0.71 (0.68)	0.305
Leptin (ng/mL)^a^	10.9 (7.2)	12.5 (12.6)	0.377	18.7 (16.1)	14.9 (15.5)	0.022
Adiponectin (μg/mL)^a^	6.45 (3.75)	6.37 (3.87)	0.507	7.46 (2.77)	7.92 (3.14)	0.248
Leptin/adiponectin ratio^a^	2.87 (3.02)	3.66 (4.82)	0.220	3.16 (3.40)	2.20 (2.04)	0.013

Data are presented as the mean (standard deviation). *p*-values were obtained by paired *t*-test or ^a^Wilcoxon signed-rank test.

We measured the changes in serological biomarkers related to metabolic status following 12 weeks of exercise in breast cancer survivors. Fasting insulin level was significantly decreased by 32% after exercise training (*p* = 0.018), whereas the change in high-sensitivity C-reactive protein level was not significant (Table 2). As an obesity-related adipokine, the serum level of leptin was decreased by exercise training (*p* = 0.022), whereas leptin levels remained unchanged in the control group. The leptin-to-adiponectin ratio, a preferential index of cardiometabolic diseases, was significantly reduced by exercise training (*p* = 0.013), although the adiponectin level remained unchanged (Table 2).

**Fig 2:**
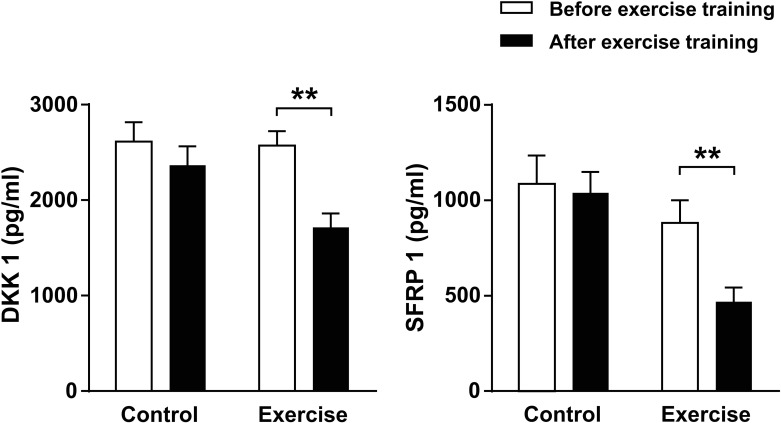
Changes in circulating levels of Dickkpof-1 (DKK1) and secreted frizzled-related protein-1 (SFRP1) elicited by 12-weeks of exercise training in breast cancer survivors. **p < 0.01 according to Wilcoxon signed-rank test.

We performed an enzyme linked immune sorbent assay to measure the serum levels of DKK1 and SFRP1, which are negative modulators of the Wnt signaling pathway. As shown in Fig 2, exercise training elicited noteworthy reductions in DKK1 (baseline vs. after 12 weeks, mean ± SEM, 2571 ± 151 vs. 1716 ± 145 pg/ml, *p* = 0.002) and SFRP1 (880 ± 119 vs. 468 ± 75 pg/ml, *p* = 0.008) levels in the serum of breast cancer survivors, whereas no changes were detected in the control group (DKK1: 2613 ± 203 vs. 2365 ± 197 pg/ml, *p* = 0.08; SFRP1: 1,085 ± 149 vs. 1,039 ± 110 pg/ml, *p* = 0.35). Additionally, we measured serum level of DKK1 in twenty-one healthy volunteers. We observed that basal DKK1 levels of the age-matched healthy women were significantly lower than those of breast cancer survivors (healthy women vs. breast cancer survivors, 1831 ± 161 vs. 2594 ± 128 pg/ml, *p* < 0.001). However, exercise did not affect the serum level of DKK1 in the healthy women.

## Discussion

Exercise, especially combined aerobic-resistance exercise regimen, not only improves body composition and cardio-respiratory fitness, but also enhances musculoskeletal strength and range of motion of the shoulder in breast cancer survivors [30–32]. We adopted a combined exercise regimen to test whether the beneficial effects of exercise training occur in Wnt signaling-related biomarkers as well. In this study, we evaluated the effects of long-term exercise training on parameters related to physical fitness and serological markers in survivors of breast cancer. Exercise training for 12 weeks successfully improved muscle strength, endurance, and flexibility. Body fat was reduced and insulin sensitivity was enhanced by the exercise program. We analyzed the changes in arm circumference as a functional characteristic. Patients and survivors of breast cancer present declines in physical performance and edema of the shoulder and arm. Additionally, they have insufficient functional shoulder range of motion after surgery [33]. It has been indicated that various exercises enhance shoulder movement and arm edema [34]. Our study showed that arm circumferences were significantly decreased in the exercised group, which may contribute to improved functional movements in survivors of breast cancer.

These beneficial effects of exercise were accompanied by reduced serum levels of the Wnt signaling modulators DKK1 and SFRP1. Over the past 20 years, various regulatory factors in Wnt signaling have been suggested and the impacts of their serum levels have been investigated. DKK1 was proposed as an independent prognostic biomarker of relapse-free survival in patients with breast cancer [16]. A previous study demonstrated that the serum level of DKK1 in breast cancer was correlated with tumor grade and lymph node metastasis. Furthermore, circulating DKK1 level is consistently increased during bone metastases in patients with breast cancer [22]. Hence, regulating DKK1 function or expression was suggested as an important therapeutic target in tumor-induced bone resorption and multiple myeloma [35,36].

Currently, the mechanism for regulation of tumor progression by Wnt inhibitors is not clearly understood. However, it has been suggested that downregulation or deletion of β-catenin in myeloid-derived cells induces immunosuppression and tumor cell growth [37]. Increased serum level of DKK1, an inhibitor of the Wnt-β-catenin pathway, has been reported in pancreatic, gastric, hepatic, lung, esophagus, and breast cancer patient [38]. Furthermore, high circulating DKK1 levels correlate with poor prognosis in various cancers [39]. D’Amico et al. reported that percentage of CD4+ and CD8+ T cells are increased in anti-DKK1 treated mice. The authors showed that anti-DKK1 treatment increased β-catenin expression in myeloid-derived suppressor cells and suggested that attenuating DKK1 activity by neutralization recruited T cells to the tumor site and decreased tumor growth [25]. These results support our hypothesis that reduction of DKK1 could contribute to the combat against cancer progression.

To date, only two studies have shown an effect of exercise on the expression levels of DKK1. In a study on humans, reduced serum level of DKK1 was detected 3 days after the subject completed an ultra-marathon (246 km) compared to pre- and immediate post-race levels of DKK1 in healthy individuals [26]. In an experimental animal study, sedentary rats showed higher expression levels of DKK1 compared to controls [27]. Wnt expression is also increased in exercised human subjects and animal models [40,41]. In addition, it has been reported that increased Wnt proteins regulate effector T‑cell development, regulatory T‑cell activation and dendritic-cell maturation [42]. In addition, Gattinoni et al. suggested that canonical Wnt signaling stimulates proliferation of immature T and B cells [43]. Therefore, activation of Wnt signaling in immune cells by exercise may stimulate cancer immune response leading to tumor suppression. Moreover, Cao Dinh et al. observed that exercise is known to increase CD4+/CD8+ counts and improve immune functions [44]. Our hypothesis suggests that a diminution of Wnt inhibitor by exercise could have immune-stimulatory effects to prevent tumor growth and cancer stage progression.

This is the first study showing that exercise training leads to decreased serum SFRP1 levels in patients with cancer. Accumulating studies have indicated that aberrant and frequent methylation and silencing of the SFRP1 gene results in activation of the Wnt pathway in breast carcinogenesis. However, the availability of SFRP1 expression as a prognostic marker is highly limited in early stage of cancer, as SFRP1 expression does not reflect the tumor grade or lymph node metastasis status associated with poor prognosis [45–47]. Interestingly, SFRP1 was found to be expressed in mature adipocytes in human adipose tissue and was increased in obese subjects [48]. In addition, SFRP1 may act as a determinant of adipose tissue expandability by participating in the paracrine regulation of human adipogenesis. In this regard, decreased serum SFRP1 by exercise training appears to be attributable to improved body composition, including decreases in body fat percentage and visceral fat areas.

Breast cancer is closely associated with metabolic syndrome [49,50]. The serum levels of various adipokines, including leptin and adiponectin, have been indicated as biomarkers for insulin resistance and metabolic syndrome [51]. Leptin plays a key role in the control of food intake, metabolism, and energy consumption. However, metabolic stress induces leptin resistance and increases the leptin/adiponectin ratio. To date, accumulating evidence indicates that increased serum levels of leptin and decreased adiponectin are associated with breast cancer risk [50,51]. In contrast, weight loss with exercise decreases the leptin/adiponectin ratio [52,53]. Our results also revealed significant decreases in both the serum level of leptin and leptin/adiponectin ratio. However, other studies indicated that 6-month combined exercise intervention programs did not affect the plasma level of adiponectin, which was similar to our results [33,34]. Increased serum levels of insulin caused by insulin resistance induce proliferative abnormalities, which are likely to cause breast cancer [54]. Moreover, increased insulin levels are associated with greater estrogen production, which can have negative effects on breast cancer. Therefore, reducing the plasma level of insulin through exercise can also have therapeutic effects on survivors of breast cancer.

Our study had several limitations. First, this was a pilot study. Pilot studies have inherent limitations such as small sample sizes. The small sample size reduced the statistical power in identifying significant changes. In addition, the duration of the exercise intervention program may not be sufficiently long and may have influenced the variables related to pre- and post-exercise intervention.

## Conclusion

We demonstrated that long-term exercise decreases the serum levels of DKK1 and SFRP1 and improves physical fitness and biomarker levels related to metabolic conditions in breast cancer survivors. These results indicate the possible involvement of Wnt signaling in the beneficial effects of exercise in patients with cancer. Further studies are required to elucidate the molecular mechanisms of these changes under pathological conditions as well as during interventional periods. These efforts will reveal useful diagnostic/prognostic biomarkers and therapeutic targets related to exercise-induced benefits in patients with cancer.

## Supporting information

S1 Text Study protocol (Original Korean version). (DOCX)

S2 Text Study protocol (English version). (DOCX)

S1 Table CONSORT checklist. (DOCX)
