# Supplementary material for: Effects of exercise training on circulating levels of Dickkpof-1 and secreted frizzled-related protein-1 in breast cancer survivors: A pilot single-blind randomized controlled trial
Source: PLoS One. 2017 Feb 8;12(2):e0171771. doi: 10.1371/journal.pone.0171771 (PMC5298304; doi:10.1371/journal.pone.0171771)
Supplement: S1 Text — (DOCX) [file pone.0171771.s001.docx]

*Department of Physiology and Hematology-Oncology*

**운동훈련이 유방암 생존자의 건강관련 체력 및 생체지표에 미치는 영향**

**서론**

유방암 환자 및 생존자들은 다양한 항암치료의 부작용으로 인한 신체 능력의 저하 및 급성 만성 통증, 피로, 관절 가동범위의 손실 등의 다양한 합병증들이 나타낸다^1-2.^ 규칙적인 운동은 신체활동능력을 향상시키고 항암치료에 따르는 다양한 부작용을 완화시킴으로 삶의 질을 향상에 기여한다. 따라서 항암 치료기간이나 치료 후, 지속적인 운동참여가 권장된다**^2-4^**. 일반적으로 운동이 암환자에게 가져다 주는 다양한 이점들은 알려져 있으나, 암의 발병과 진행 및 그에 따른 기전들은 여전히 분명하지 않다. WNT 신호전달체계 (Wingless and integration site growth factor)는 종양형성과 관련된 주요한 신호전달체계 중 하나로써**^5^** WNT의 분비 및 그 후속 반응기 (downstream effector)는 종양성장 및 전이의 중요한 조절인자로 알려진다**^6^.** 또한 WNT 및 WNT 후속 반응기의 돌연변이는 유방암을 비롯한 다양한 암과의 연관성을 나타낸다**^5,6^**. 특히 유선의 발암조직에서는 WNT 신호 전달체계의 조절장애가 관찰된다**^7-9^**. 최근 20년간 WNT 신호체계에 대한 다양한 조절인자들이 연구되어 왔다. 그 중, Dickkpof-1 (DKK1)은 low-density lipoprotein receptor-related protein 5 and 6 (LRP5/6) 및 Kremen 단백질과 결합체를 형성하여 endocytosis를 통한 β*-*catenin의 proteosomal degradation을 유발하여 WNT 신호전달체계를 막는 가용성 억제제로 알려진다^9-11^. 또한 Secreted Frizzled-related protein-1 (SFRP1)는 Wnt ligands 나 Frizzled receptors에 결합하여 WNT 신호전달체계를 억제하는 것으로 알려진다^9^. 그러나 최근 유방암 환자가 건강한 여성이나 유방암 완치 대상자에 비해 혈중 DKK1가 높다는 역설적인 결과가 보고되었다**^12,13^**. 높은 수준의 혈중 DKK1은 유방암의 골 전이 및 그 사망률과의 연관성이 제시되기도 하였다^10,11^. 한편, 뼈에 대한 전이파종과 무관하게 췌장암, 전립선암, 위암, 간암 및 폐암 환자의 높은 혈청 내 DKK1 수준과 그들의 좋지 못한 예후간의 연관성이 관찰된다 ^14-16^. 더욱이 DKK1의 중화를 통한 억제작용은 암의 성장을 감소시키는 것으로 알려진다**^16^**. 그러므로, DKK1의 조절은 항암 및 예후개선에 중요한 치료적 표적이 될 수 있을 것으로 사료된다. 최근 보고에 따르면 Ultra-distance marathon 후에 혈중 DKK1의 감소가 보고되었다**^17^**. 또한 좌식생활의 동물모델에서 뇌조직의 높은 DKK1 발현을 보임으로서 간접적으로 신체활동에 의한 DKK1의 하향조절을 시사한바 있다**^18^**.

**연구설계**

***연구대상자***

원주기독병원 종양내과 내원한 유방암 생존자들로서 2014년 6월 1일부터 2014년12월 31일까지 모집한다. 치료이력과 월경상태는 참여자 동의 하에 혈액종양센터의 임상기록을 활용하며 연구에 포함된 모든 참여자에게 동의서를 받는다. 전문 종양학자의 의학적 승인과 다음 제시한 기준들에 부합하는 대상자가 본 연구에 참여한다.

- 참여기준:
- 유방암 진단 분류기준인 TNM (Tumor Node Metastasis)분류에 따른 I-III기의 대상자
- 종양절제술 또는 유방 제거수술을 경험한자 여성
- 수술 후 항암요법이나 방사선 요법이 종료되고 운동프로그램에 참여가능 자
- 비흡연자 (i.e.,12개월이상 금연자)
- 혈액종양 전문의로부터 12주 운동프로그램에 참여가 가능하다고 판단된 자
- 제외기준:
- 조절되지 않는 고혈압 및 당뇨를 포함한 만성질환의 이력
- 최근 6개월이내 10%이상의 체중 감소
- 전이성의 질환
- 6개월이내 하루 60분 이상의 규칙적인 운동에 참여한 자
- 심장혈관계, 호흡계, 근골격계 질환이나 관절에 문제로 중등강도의 운동이 불가능한 자

***연구절차***

인구통계확적 특성은 자가설문지를 통해 확인하며, 측정검사는 운동12주 전후로 신체계측, 건강관련체력, 신체조성 및 채혈검사 등이 수행된다. 사전검사 후에, 참가자는 밀봉된 표식과 컴퓨터를 활용하여 1:1무선배정하여 운동 중재군이나 대조군으로 분류한다. 측정자들은 운동군과 대조군의 그룹할당을 모르는 상태에서 측정검사를 진행한다. 통계분석 완료 전까지 통계 분석자 역시 그룹할당을 모르는 상태를 유지한다. 그룹배정에 대한 참여자 암맹은 실시하지 않으나, 측정검사자 들로 하여금 어떤 항목도 관찰되어지지 않도록 자신의 경험을 함구하도록 교육한다. 모든 연구과정은 연세대학교 원주의과대학의 연구윤리위원회에 심의를 거쳐 승인되었다 (YWMR-14-0-042).

***운동군***

운동프로그램은 미국스포츠의학회의 암환자 운동 지침에 따라 설계되었고, 12주 동안 운동전문가 관리감독 하에 시행된다. 운동의 강도는 개인의 운동자각도 (RPE) 에 따라 점증적으로 증가시키며 초기 4주간은 RPE 11~13에서, 4주이후는 RPE 13~15 수준으로 설정한다. 각 운동세션은 어깨의 강직과 제한된 관절가동범위 완화를 위한 전신 스트레칭, climbing, shoulder glides (inferior, anterior, and posterior), 어깨 진자 운동을 10분간 시행한다. 본 운동은 17-cm (6.7-inch) platform의 Step box를 통한 유산소운동을 20분간 시행하며, 전신근력의 증가를 위해 체중을 이용한 shoulder press, black burn exercise, wall push-up, biceps curl-up, plank exercise, leg bridge, squat, and calf raise등을20분간 시행한다. 정리운동은 가벼운 걷기 운동과 전신 스트레칭으로 10분간 실시한다.

***대조군***

대조군에게는 일상적인 신체활동을 유지하도록 안내하고 사후검사 이전에는 (12주 동안) 새로운 운동프로그램에 참여하지 않도록 권고한다. 사후검사 후 (12주 이후), 대조군 역시 운동군과 동일한 운동프로그램에 참여할 수 있는 기회를 제공한다.

***데이터분석***

수집된 모든 자료는 SPSS 22.0 software (SPSS, Inc., Chicago, IL, USA)를 이용하여 분석한다. 평균과 표준편차 또는 표준오차 등은 기술통계를 통해 산출된다. 사전-사후간의 건강관련 체력, 신체조성 및 biomarkers 의 혈중수준을 비교하기 위해 paired t-test 또는 Wilcoxon signed-rank test를 이용하여 분석하며, 통계적 유의수준은 *p* < 0.05로 설정한다.

**Ⅰ. 주요변인**

연구의 목적은 운동중재 프로그램이 건강관련 체력 및 암 관련 혈중 지표에 미치는 영향을 평가하고 이해하는데 있다. 이에 따른 측정항목은 다음과 같다.

1) 건강관련체력: 심폐지구력, 근력, 근지구력, 유연성, 민첩성, 순발력

2) 대사질환의 위험인자: 신체구성성분, 허리둘레, 혈압, 혈당, 인슐린, 혈중 지질 성분 및 C-reactive protein.

3) 암 관련 혈중 생체지표: Dickkopf-related protein 1 (DKK1), secreted frizzled-related protein 1 (SFRP1), sclerostin, osteoprotegerin, osteopontin, growth differentiation factor 15 (GDF-15), insulin like growth factor 1 (IGF-1), and IGFBP-3.

***방법***

1. 건강관련체력 측정

심폐지구력의 측정은 multi-stage 20 meters shuttle run test (최대반복횟수)를 이용하여 측정한다. 근력은 악력을 이용하여 최대근력(kg)을 측정하고 근지구력은 sit-up test를 이용하여 1분간 최대반복 횟수를 측정한다. 근 파워는 멀리뛰기 (최대 수평거리) 로 측정하고 2회 반복하여 최대치를 기록한다. 민첩성은 10M 왕복달리기 검사 시 소요시간을 측정하여 활용하여 유연성은 앉아 앞으로 윗몸 굽히기 (cm) 를 이용하여 측정한다.

2. 신쳬계측 및 신체구성성분

체중과 신장은 각각 0.1 kg와 0.1 cm 단위로 측정한다. 허리둘레는 (cm) 하부 갈비뼈와 엉덩이뼈 사이의 능선을 측정한다. 체질량지수는 체중/신장 (kg/m^2^)로 계산한다. 체지방량(kg)과 근육량(kg)은 bio-impedance analyzer를 통해 측정하고 체지방률은 체중 대비 체지방량의 비율로 계산한다.

3. Biomarker 분석

채혈는 전완주정맥에서 이뤄지며 혈청분리관을 이용하여 혈청을 추출한다. 채취된 혈액은 3000 rpm (1,000 ×g)에서 10분간 원심분리 후 혈청을 추출하여 즉시 영하 80°C에 보관된다. WNT 신호전달관련 물질인 DKK1, SFRP1, sclerostin의 분석은 ELISA (enzyme linked immune sorbent assay) Kit를 통해 분석한다.

- DKK1, minimal detectable density, 15.6 pg/ml; standard curve range, 31.2 - 2,000 pg/ml(R&D systems)
- SFRP1, minimal detectable density, 53 pg/ml; standard curve range, 156 - 10,000 pg/ml (USCN Life Science Inc.)
- Sclerostin, minimal detectable density, 6.96 pg/ml; standard curve range, 7.49 - 1,820 pg/ml (R&D systems)

**Ⅱ. 보조변인**

혈중 Adipokine, Inflammatory cytokine, 종양관련 cytokine들은 보조변인으로 측정한다. 운동프로그램

의 종료 후 종양관련인자들의 변화와 그 관계들을 분석할 계획이다.

***실험방법***

1. 혈중 Adipokine들의 변화

Leptin과 Adiponectin의 혈중 농도는 ELISA kit (R&D systems) 을 이용하여 측정

- Leptin, minimal detectable density, 7.8 pg/ml; standard curve range, 15.6 - 1,000 ng/ml
- Adiponectin, minimal detectable density, 0.891 ng/ml; standard curve range, 3.9 - 250 ng/ml

2. 혈중 염증지표들의 변화

IL-1 beta, IL-10, IL-11, TNF-alpha 의 혈중 농도는 commercial luminex multiplexed cytokine assay panels (R&D systems) 을 이용하여 측정

- IL-1 beta, minimal detectable density, 0.8 pg/ml; standard curve range, 17.8 - 4,320 pg/ml
- IL-10, minimal detectable density, 1.6 pg/ml; standard curve range, 13.7 - 3,340 pg/ml
- IL-11, minimal detectable density, 24.7 pg/ml; standard curve range, 0.5 - 125.4 ng/ml
- TNF-alpha, minimal detectable density, 1.2 pg/ml; standard curve range, 14 - 3,410 pg/ml

3. 혈중 종양관련인자들의 변화

Osteosteoprotegerin, osteopontin, growth differentiation factor 15 (GDF-15)의 혈중 농도는 luminex multiplexed cytokine assay panels (R&D systems)을 이용하여 측정

- Osteoprotegerin,minimal detectable density, 3.62 pg/ml; standard curve range, 81.4 - 19,770 pg/ml
- Osteopontin, minimal detectable density, 413 pg/ml; standard curve range, 3.4 - 826.9 ng/ml
- GDF-15, minimal detectable density, 1.2 pg/ml; standard curve range, 34 - 8,270 pg/ml

4. 혈중 인슐린 및 C-reactive protein의 변화

insulin and high-sensitivity와 C-reactive protein 의 혈중 농도는 electrochemiluminescence immunoassay (Roche cobas 8000-e602 module, Roche Diagnostics, Basel, Switzerland)와 latex-enhanced immunoturbidimetric assay (Roche-Hitachi cobas c system, Roche Diagnostics)을 이용하여 각각 측정

**REFERENCES**

1. J. M. Binkley *et al.*, Patient perspectives on breast cancer treatment side effects and the prospective surveillance model for physical rehabilitation for women with breast cancer. *Cancer***118**, 2207-2216 (2012).

2. P. D. Loprinzi, B. J. Cardinal, Effects of physical activity on common side effects of breast cancer treatment. *Breast Cancer***19**, 4-10 (2012).

3. R. Knols, N. K. Aaronson, D. Uebelhart, J. Fransen, G. Aufdemkampe, Physical exercise in cancer patients during and after medical treatment: a systematic review of randomized and controlled clinical trials. *J Clin Oncol***23**, 3830-3842 (2005).

4. M. L. McNeely *et al.*, Effects of exercise on breast cancer patients and survivors: a systematic review and meta-analysis. *CMAJ***175**, 34-41 (2006).

5. P. Polakis, Wnt signaling in cancer. *Cold Spring Harb Perspect Biol***4**, (2012).

6. J. N. Anastas, R. T. Moon, WNT signalling pathways as therapeutic targets in cancer. *Nat Rev Cancer***13**, 11-26 (2013).

7. K. R. Brennan, A. M. Brown, Wnt proteins in mammary development and cancer. *J Mammary Gland Biol Neoplasia***9**, 119-131 (2004).

8. T. Schlange, Y. Matsuda, S. Lienhard, A. Huber, N. E. Hynes, Autocrine WNT signaling contributes to breast cancer cell proliferation via the canonical WNT pathway and EGFR transactivation. *Breast Cancer Res***9**, R63 (2007).

9. R. Surana *et al.*, Secreted frizzled related proteins: Implications in cancers. *Biochim Biophys Acta***1845**, 53-65 (2014).

10. S. J. Zhou, S. R. Zhuo, X. Q. Yang, C. X. Qin, Z. L. Wang, Serum Dickkopf-1 expression level positively correlates with a poor prognosis in breast cancer. *Diagn Pathol***9**, 161 (2014).

11. T. D. Rachner, A. Gobel, P. Benad-Mehner, L. C. Hofbauer, M. Rauner, Dickkopf-1 as a mediator and novel target in malignant bone disease. *Cancer Lett***346**, 172-177 (2014).

12. N. Voorzanger-Rousselot *et al.*, Increased Dickkopf-1 expression in breast cancer bone metastases. *Br J Cancer***97**, 964-970 (2007).

13. N. Voorzanger-Rousselot, F. Journe, V. Doriath, J. J. Body, P. Garnero, Assessment of circulating Dickkopf-1 with a new two-site immunoassay in healthy subjects and women with breast cancer and bone metastases. *Calcif Tissue Int***84**, 348-354 (2009).

14. S. X. Han *et al.*, Serum dickkopf-1 is a novel serological biomarker for the diagnosis and prognosis of pancreatic cancer. *Oncotarget***6**, 19907-19917 (2015).

15. T. D. Rachner *et al.*, High serum levels of Dickkopf-1 are associated with a poor prognosis in prostate cancer patients. *BMC cancer***14**, 649 (2014).

16. L. D'Amico *et al.*, Dickkopf-related protein 1 (Dkk1) regulates the accumulation and function of myeloid derived suppressor cells in cancer. *The Journal of experimental medicine***213**, 827-840 (2016).

17. K. Kerschan-Schindl *et al.*, Changes in Serum Levels of Myokines and Wnt-Antagonists after an Ultramarathon Race. *PLoS One***10**, e0132478 (2015).

18. S. Bayod *et al.*, Wnt pathway regulation by long-term moderate exercise in rat hippocampus. *Brain Res* **1543**, 38-48 (2014).
