# Supplementary material for: Effects of exercise training on circulating levels of Dickkpof-1 and secreted frizzled-related protein-1 in breast cancer survivors: A pilot single-blind randomized controlled trial
Source: PLoS One. 2017 Feb 8;12(2):e0171771. doi: 10.1371/journal.pone.0171771 (PMC5298304; doi:10.1371/journal.pone.0171771)
Supplement: S2 Text — (DOCX) [file pone.0171771.s002.docx]

*Department of Physiology and Hematology-Oncology*

**Effects of exercise training on health-related physical fitness and biomarkers in breast cancer survivors**

**INTRODUCTION**

Patients and survivors of breast cancer present impaired physical fitness and various complications including acute and chronic pain, severe fatigue, limited range of motion, and bone loss attributable to anticancer treatments**^1,2^**. Therefore, regular exercise during and following cancer treatments has been recommended to enhance physical capabilities and relieve side-effect severities, leading to an improved quality of life**^2-4^**. Despite the known general benefits to patients with cancer, the effects of exercise on the initiation and progression of the tumor itself remain unclear.

Wingless and integration site growth factor (Wnt) signaling is one of the major tumorigenesis-related signaling pathways**^5^**. Secreted Wnts and their downstream effectors regulate critical processes during tumor growth and metastasis**^6^**. Additionally, their mutations are associated with breast cancer and other common types of cancer**^5,6^**. Dysregulated Wnt signaling has been detected in carcinogenesis of the mammary gland**^7-9^**. For the past two decades, a number of endogenous modulators of Wnt signaling have been identified. Among them, Dickkpof-1 (DKK1) is a soluble inhibitor of the Wnt signaling pathway, by binding to low-density lipoprotein receptor-related protein 5 and 6 and Kremen protein to induce endocytosis, causing proteosomal degradation of β*-*catenin**^9-11^**. Secreted Frizzled-related protein-1 (SFRP1) blocks Wnt signaling by binding to Wnt ligands or Frizzled receptors**^9^**. Paradoxically, DKK1 levels were found to be markedly increased in patients with breast cancer compared to in women in complete remission and healthy controls**^12,13^**. Higher serum levels of DKK1 were correlated with bone metastasis of breast cancer and its mortality**^10,11^**. A correlation between high DKK1 and poor prognosis was also observed in serologic samples from patients with pancreas, prostate, stomach, liver, and lung cancers, regardless of the presence of metastatic dissemination to the bone**^14-16^**. Furthermore, inhibition of DKK1 by neutralization decreases tumor growth**^16^**. Therefore, the regulation of DKK1 may be a therapeutic target in the development of anticancer therapy. A recent study reported that serum DKK1 levels were significantly decreased after participation in an ultradistance marathon**^17^**. An animal study also showed that sedentary conditions elevated DKK1 expression in brain tissue, indirectly showing that physical activity downregulates DKK1**^18^**.

**STUDY DESIGN**

***STUDY PARTICIPANTS***

Survivors of breast cancer, who visited the hemato-oncology center of Wonju Severance Christian Hospital, are recruited between June 1 and December 31, 2014. Treatment history and menstrual status are obtained from the clinical reports of the hemato-oncology center with the consent of participants. Informed consent will be obtained from all participants included in the study.

They are eligible to participate in this study if they met the following inclusion criteria without exclusion criteria, with medical clearance from their oncologist

- Inclusion Criteria:
- Have diagnosed as a stage of I-III breast cancer
- Have undergone a lumpectomy or mastectomy
- Have completed neoadjuvant/adjuvant chemotherapy and able to initiate Exercise program
- Nonsmokers (i.e., not smoking during previous 12 months)
- Able to provide physician clearance to participate in exercise program for 12 weeks
- Exclusion Criteria:
- History of chronic disease including diabetes, uncontrolled hypertension or thyroid disease
- Weight reduction >= 10% within past 6 months
- Metastatic disease
- Participate in more than 60 minutes of exercise per week in the past 6 months
- Cardiovascular, respiratory or musculoskeletal disease or joint problems that preclude moderate physical activity

***STUDY PROCEDURES***

Demographic characteristics are collected through self-reports. Measurements are conducted at baseline and 12 weeks post-exercise training, which include anthropometry, body composition, health–related fitness levels, and blood sample collection. Following baseline assessments, the eligible participants are randomly assigned to either an exercise intervention group or a control group using sealed, computer generated random numbers with the allocation ratio of 1 to 1. Four research staff members who are unaware of group assignment performed all outcome assessments. The statistician is unaware of treatment allocation until completion of the statistical analyses. Participants are not blinded to their assignment but are unaware of main outcome measures and are instructed to avoid mentioning anything regarding their study experience to the assessors. All procedures were reviewed and approved by the Medical Ethics Committee of Yonsei University Wonju College of Medicine, Korea (YWMR-14-0-042).

***EXERCISE GROUP***

The exercise training program is designed following the American College of Sports Medicine cancer guidelines (exercise intensity, frequency, time, and type), and are performed at least thrice weekly for 12 weeks, under the direct supervision of exercise physiologists. Exercise training is conducted with individual’s ratings of perceived exertion intensities within 11–13 that are gradually and moderately increased at 4-week intervals until reaching a rating of 13–15. Each session begins with a warm-up consisting whole body stretching and flexibility exercises for shoulder muscles stiffness such as finger climbing, shoulder glides (inferior, anterior, and posterior), and pendulum exercises for 10 min. The exercise program incorporates step aerobics on 17-cm (6.7-inch) platforms for 20 min followed by the strength training using body weight consisting of shoulder press, black burn exercise, wall push-up, biceps curl-up, plank exercise, leg bridge, squat, and calf raise for 20 min. At the end of the session, subjects perform cool-down involving easy walking and stretching exercises for 10 min.

***CONTROL GROUP***

Participants in the control group are instructed to maintain their routine physical activities and not to participate any new exercise programs during the 12-weeks study period. Afterward, the subjects who complete both pre and post-test received an opportunity to participate in the same exercise program that intervention group has performed.

***DATA ANALYSIS***

All data are analyzed using SPSS 22.0 software (SPSS, Inc., Chicago, IL, USA). Descriptive statistics are calculated to identify the means and standard deviations or standard errors of mean (SEM). Paired t-test or Wilcoxon signed-rank test are used to compare the levels of health-related fitness, body composition, and serum biomarkers between before and after training. Statistical significance is set at p < 0.05.

**Ⅰ. PRIMARY OUTCOMES**

The aim of the study is to assess and understand the effects of an exercise program on health-related physical fitness and biomarkers involving cancer-related molecules. For this reasons we will evaluate:

1) Components of health-related physical fitness including cardiorespiratory fitness, muscular exercise capacity and flexibility.

2) Risk parameters of metabolic diseases including body composition, waist circumference, blood pressure and circulating levels of glucose, insulin, lipids components and C-reactive protein.

3) Changes of cancer-related biomarkers such as serum levels of dickkopf-related protein 1 (DKK1), secreted frizzled-related protein 1 (SFRP1), sclerostin, osteoprotegerin, osteopontin, growth differentiation factor 15 (GDF-15), insulin like growth factor 1 (IGF-1), and IGFBP-3.

***METHODS***

1. Health-related physical fitness measurements

Aerobic capacity is assessed using multi-stage 20 meters shuttle run test (the maximum number of repetitions). Muscular endurance is assessed using sit-up test for 30 seconds (the maximum number of repetitions). Muscular strength is assessed by the maximum voluntary strength of handgrip (kg). Muscular power is assessed using standing long jump test (the maximum horizontal distance of two trials, cm). Agility is assessed using 10 meters agility shuttle run test (the time taken to complete a 10 meters course is recorded, seconds). Flexibility is assessed using sit and reach test (the greater distance of two trials, cm)

2. Anthropometry and body composition measurements

Body weight and height are measured to the nearest 0.1 kg and 0.1 cm, respectively. Waist circumference is measured at the midpoint between the lower rib margin and the iliac crest (expressed in cm). Body mass index is calculated as body weight / height (kg per square meters). Body fat and lean mass are measured by a bio-impedance analyzer (expressed as kg). Then, percentage body fat is calculated as body fat mass (kg) divided by weight (kg).

3. Biomarker measurements

Blood samples are drawn from the antecubital vein and collected in serum separation tubes. The tubes are centrifuged at 3000 rpm (1,000 ×g) for 10 min, and serum samples are collected and immediately stored at -80°C until analysis. Commercially available enzymelinked immune sorbent assay (ELISA) kits are used to measure the changes of serum levels of Wnt signaling-related molecules such as DKK1, SFRP1 and sclerostin.

- DKK1, minimal detectable density, 15.6 pg/ml; standard curve range, 31.2 - 2,000 pg/ml (R&D systems)
- SFRP1, minimal detectable density, 53 pg/ml; standard curve range, 156 - 10,000 pg/ml (USCN Life Science Inc.)
- Sclerostin, minimal detectable density, 6.96 pg/ml; standard curve range, 7.49 - 1,820 pg/ml (R&D systems)

**Ⅱ. SECONDARY OUTCOMES**

The serum levels of adipokines, inflammatory and cancer-related cytokines are measured as secondary outcome measures. After which, we will examine the relationship between the changes of each biomarker including cancer-related molecules elicited by an exercise program.

***METHODS***

1. Changes of serum levels of adipokines.

The serum concentration of leptin and adiponectin are measured by commercial ELISA kits (R&D systems).

- Leptin, minimal detectable density, 7.8 pg/ml; standard curve range, 15.6 - 1,000 ng/ml
- Adiponectin, minimal detectable density, 0.891 ng/ml; standard curve range, 3.9 - 250 ng/ml

2. Changes of serum levels of inflammatory-related cytokines

The serum concentration of IL-1 beta, IL-10, IL-11 and TNF-alpha are measured using commercial luminex multiplexed cytokine assay panels (R&D systems).

- IL-1 beta, minimal detectable density, 0.8 pg/ml; standard curve range, 17.8 - 4,320 pg/ml
- IL-10, minimal detectable density, 1.6 pg/ml; standard curve range, 13.7 - 3,340 pg/ml
- IL-11, minimal detectable density, 24.7 pg/ml; standard curve range, 0.5 - 125.4 ng/ml
- TNF-alpha, minimal detectable density, 1.2 pg/ml; standard curve range, 14 - 3,410 pg/ml

3. Changes of serum levels of cancer-related molecules.

The serum concentrations of osteoprotegerin, osteopontin and growth differentiation factor 15 (GDF-15) are measured by commercial luminex multiplexed cytokine assay panels(R&D systems).

- Osteoprotegerin, minimal detectable density, 3.62 pg/ml; standard curve range, 81.4 - 19,770 pg/ml
- Osteopontin, minimal detectable density, 413 pg/ml; standard curve range, 3.4 - 826.9 ng/ml
- GDF-15, minimal detectable density, 1.2 pg/ml; standard curve range, 34 - 8,270 pg/ml

4. Serum insulin and high-sensitivity C-reactive protein are measured by electrochemiluminescence immunoassay (Roche cobas 8000-e602 module, Roche Diagnostics, Basel, Switzerland) and latex-enhanced immunoturbidimetric assay (Roche-Hitachi cobas c system, Roche Diagnostics), respectively.

**REFERENCES**

1. J. M. Binkley *et al.*, Patient perspectives on breast cancer treatment side effects and the prospective surveillance model for physical rehabilitation for women with breast cancer. *Cancer***118**, 2207-2216 (2012).

2. P. D. Loprinzi, B. J. Cardinal, Effects of physical activity on common side effects of breast cancer treatment. *Breast Cancer***19**, 4-10 (2012).

3. R. Knols, N. K. Aaronson, D. Uebelhart, J. Fransen, G. Aufdemkampe, Physical exercise in cancer patients during and after medical treatment: a systematic review of randomized and controlled clinical trials. *J Clin Oncol***23**, 3830-3842 (2005).

4. M. L. McNeely *et al.*, Effects of exercise on breast cancer patients and survivors: a systematic review and meta-analysis. *CMAJ***175**, 34-41 (2006).

5. P. Polakis, Wnt signaling in cancer. *Cold Spring Harb Perspect Biol***4**, (2012).

6. J. N. Anastas, R. T. Moon, WNT signalling pathways as therapeutic targets in cancer. *Nat Rev Cancer***13**, 11-26 (2013).

7. K. R. Brennan, A. M. Brown, Wnt proteins in mammary development and cancer. *J Mammary Gland Biol Neoplasia***9**, 119-131 (2004).

8. T. Schlange, Y. Matsuda, S. Lienhard, A. Huber, N. E. Hynes, Autocrine WNT signaling contributes to breast cancer cell proliferation via the canonical WNT pathway and EGFR transactivation. *Breast Cancer Res***9**, R63 (2007).

9. R. Surana *et al.*, Secreted frizzled related proteins: Implications in cancers. *Biochim Biophys Acta***1845**, 53-65 (2014).

10. S. J. Zhou, S. R. Zhuo, X. Q. Yang, C. X. Qin, Z. L. Wang, Serum Dickkopf-1 expression level positively correlates with a poor prognosis in breast cancer. *Diagn Pathol***9**, 161 (2014).

11. T. D. Rachner, A. Gobel, P. Benad-Mehner, L. C. Hofbauer, M. Rauner, Dickkopf-1 as a mediator and novel target in malignant bone disease. *Cancer Lett***346**, 172-177 (2014).

12. N. Voorzanger-Rousselot *et al.*, Increased Dickkopf-1 expression in breast cancer bone metastases. *Br J Cancer***97**, 964-970 (2007).

13. N. Voorzanger-Rousselot, F. Journe, V. Doriath, J. J. Body, P. Garnero, Assessment of circulating Dickkopf-1 with a new two-site immunoassay in healthy subjects and women with breast cancer and bone metastases. *Calcif Tissue Int***84**, 348-354 (2009).

14. S. X. Han *et al.*, Serum dickkopf-1 is a novel serological biomarker for the diagnosis and prognosis of pancreatic cancer. *Oncotarget***6**, 19907-19917 (2015).

15. T. D. Rachner *et al.*, High serum levels of Dickkopf-1 are associated with a poor prognosis in prostate cancer patients. *BMC cancer***14**, 649 (2014).

16. L. D'Amico *et al.*, Dickkopf-related protein 1 (Dkk1) regulates the accumulation and function of myeloid derived suppressor cells in cancer. *The Journal of experimental medicine***213**, 827-840 (2016).

17. K. Kerschan-Schindl *et al.*, Changes in Serum Levels of Myokines and Wnt-Antagonists after an Ultramarathon Race. *PLoS One***10**, e0132478 (2015).

18. S. Bayod *et al.*, Wnt pathway regulation by long-term moderate exercise in rat hippocampus. *Brain Res***1543**, 38-48 (2014).
